# Establishment of Decision Rules and Risk Assessment Model for Preoperative Prediction of Lymph Node Metastasis in Gastric Cancer

**DOI:** 10.3389/fonc.2020.01638

**Published:** 2020-09-02

**Authors:** Chao Huang, Cegui Hu, Jinfeng Zhu, Wenjun Zhang, Jun Huang, Zhengming Zhu

**Affiliations:** Department of Gastrointestinal Surgery, The Second Affiliated Hospital of Nanchang University, Nanchang, China

**Keywords:** gastric cancer, lymph node metastasis, preoperative diagnosis, risk factors, decision rules, risk assessment model

## Abstract

**Background:** Preoperative accurate prediction of lymph node status is especially important for the formulation of treatment plans for patients with gastric cancer (GC). The purpose of this study was to establish decision rules and a risk assessment model for lymph node metastasis (LNM) in GC using preoperative indicators.

**Methods:** The clinical data of 554 patients who underwent gastrectomy with D2 lymphadenectomy were collected. A 1:1 propensity score matching (PSM) system was used, and the clinical data of the matched 466 patients were further analyzed. The important risk factors for LNM were extracted by the random forest algorithm, and decision rules and nomogram models for LNM were constructed with a classification tree and the “rms” package of R software, respectively.

**Results:** Tumor size (*OR*: 2.058; *P* = 0.000), computed tomography (CT) findings (*OR*: 1.969; *P* = 0.001), grade (*OR*: 0.479; *P* = 0.000), hemoglobin (Hb) (*OR*: 1.211; *P* = 0.005), CEA (*OR*: 1.111; *P* = 0.017), and CA19-9 (*OR*: 1.040; *P* = 0.033) were independent risk factors for LNM in GC. Tumor size did rank first in the ranking of important factors for LNM in GC and was the first-level segmentation of the two initial branches of the classification tree. The accuracy, sensitivity, specificity, and positive predictive value of the decision rules in diagnosing preoperative LNM in GC were 75.6, 85.7, 73.9, 73.5, and 79.3%, respectively. The accuracy, sensitivity, and specificity of the risk assessment model in predicting preoperative LNM in GC were 79.3, 80.3, and 79.4%, respectively.

**Conclusion:** Tumor size was the most important factor for evaluating LNM in GC. This decision rules and nomogram model constructed to take into account tumor size, CT findings, grade, hemoglobin, CEA, and CA19-9 effectively predicted the incidence of LNM in preoperative GC.

## Introduction

Gastric cancer (GC) is a common malignant tumor of the digestive system with the third highest fatality rate in the world (1). Lymph node metastasis (LNM) is one of the most important prognostic factors for patients with GC (2, 3). D2 gastrectomy is a standard operation for GC, especially advanced gastric cancer (AGC) (4). For early gastric cancer (EGC) patients without LNM, endoscopic mucosal resection, and endoscopic submucosal dissection are widely accepted approaches that can not only preserve gastric function and maintain a high quality of life but also avoid post-operative complications caused by radical gastrectomy (5–7). The Japanese gastric cancer association (JGCA) proposed that for differentiated T1a EGC without LNM, endoscopic resection or partial resection plus D1/D1+ lymphadenectomy should be considered, but standard D2 lymphadenectomy must be performed in the presence of LNM (8). The National Comprehensive Cancer Network (NCCN) guidelines recommend perioperative chemotherapy or preoperative chemoradiotherapy for lymph node-positive AGC (9). The Japanese gastric cancer treatment guidelines 2018 (5th edition) also proposed that neoadjuvant chemotherapy (NAC) should be required for GC patients who have extensive and large-volume LNM (10). Tsuburaya et al. (11) showed that NAC followed by radical gastrectomy could improve the survival rate of patients with AGC with a large number of metastatic lymph nodes along the major perigastric vessels and/or aorta. Sasako et al. (12) found that radical gastrectomy for GC patients with para-aortic LNM did not improve patient survival times. Moreover, the benefits of NAC have been confirmed in some randomized controlled trials, and most oncologists recommend that NAC should be given to patients with AGC, especially those with LNM, to reduce preoperative TNM staging, thereby improving the rate of radical resection (13, 14). Therefore, accurate preoperative prediction of lymph node status is crucial for the selection of a therapeutic regimen for patients with GC.

Currently, multidetector computed tomography (MDCT), endoscopic ultrasonography (EUS), magnetic resonance imaging (MRI), and positron emission tomography-computed tomography (PET-CT) are used to assess lymph node status in GC patients, but due to their sensitivity, specificity, and accuracy, they are controversial, especially in EGC, so they have limitations as clinical applications (15, 16). Previous studies have also identified novel molecular biomarkers that predict LNM (17, 18), but their high cost and complex techniques have limited their clinical application. Nomograms have been widely used to quantify risk factors for LNM in various cancers, including EGC (19–21), but the existing nomograms combine post-operative characteristics, thus reducing their clinical value. In this study, we used only the indicators available before surgery to establish the decision rules and risk assessment model for LNM in GC, thus providing a specific reference value for the formulation of a therapeutic schedule and the evaluation of prognosis in GC patients.

## Methods

This study was approved by the Ethics Committee of the Second Affiliated Hospital of Nanchang University and was conducted in accordance with the ethical standards of the Helsinki Declaration. All patients signed informed consent forms.

### Study Patients

The clinical data of 554 patients who underwent gastrectomy with D2 lymphadenectomy were collected in our hospital from June 2017 to April 2020. Gastroscope and contrast-enhanced MDCT were used for routine preoperative examination in all patients. In our institute, 64-slice MDCT is used. Two experienced radiologists determined the LNM by looking at axial CT images with a cross-section thickness of 5 mm. Positive was defined as the presence of any identifiable lymph nodes on the CT image. According to the presence or absence of LNM in the surgical pathological specimens, the patients were divided into an LNM group and a without LNM group. There were 312 cases in the LNM group and 242 cases in the without LNM group. According to age, sex and BMI, 1:1 matching was performed, and they clinical data of 466 patients who obtained matching were analyzed. The serum tumor marker, albumin, fibrinogen, hemoglobin (Hb), neutrophil, platelet, and lymphocyte levels were extracted at the first admission. The clinicopathological characteristics of the patients, including age, sex, BMI, tumor size, tumor location, and histological grade, were recorded in our study. Tumor size was measured according to the maximum diameter of the tumor. Laparoscopic-assisted radical gastrectomy or open radical gastrectomy was performed. Distal gastrectomy or total gastrectomy was conducted depending on the location of the tumor. The range of lymph node dissection was D2 lymphadenectomy.

### Inclusion and Exclusion Criteria

The inclusion criteria of this study were as follows: (1) patients who underwent gastrectomy with D2 lymphadenectomy; (2) no neoadjuvant radiotherapy or chemotherapy was used before surgery; and (3) there was a complete pathological report after surgery. We excluded patients with bleeding, hyperthyroidism or hypothyroidism, acute infection, no radical resection, gastric stump cancer, distant metastasis, combined with other tumors, chronic inflammation, autoimmune diseases, hematological diseases, and incomplete data.

### Statistical Analysis

The single-sample Kolmogorov-Smirnov test was used to test the normality of the data. If the quantitative data had a normal distribution, they are described as the mean ± standard deviation; otherwise, data are described as the median and quartile interval. Categorical variables are expressed as rates with 95% confidence intervals (CIs). Propensity score matching (PSM) was used to balance the covariates and number of cases between the groups. Univariate analysis between the two groups was performed using the Mann-Whitney *U*-test. Significant indicators identified in the univariate analysis were analyzed by multivariate conditional logistic regression. The random forest algorithm was used to extract the important risk factors for LNM in GC, and the mean decrease Gini (MDG) was used to rank the important indicators. The MDG quantifies which indicator contributes the most to classification accuracy (22). The cut-off value for important risk factors was determined by the receiver operating characteristic (ROC) curve analysis. The decision rules and nomogram model for LNM were constructed by classification tree and the “rms” package in R software, respectively. A classification tree is a non-linear discriminant method that uses a set of independent variables to gradually decompose the sample into smaller subgroups. This procedure iterates on each branch of the tree, and selects the independent variable with the strongest correlation with the dependent variable according to specific criteria (23). The decision rules provide specific information about risk factors based on rule induction. The ROC curve was used to evaluate the accuracy, sensitivity, and specificity of the model to predict LNM of GC. A two-sided *P* < 0.05 was considered statistically significant. Data were analyzed using SPSS 22.0 for Windows (SPSS Inc., Chicago, IL, USA) and R (version x64 3.6.1), including non-random, rpart, rpart.plot, randomForest, and rms packages.

## Results

### Clinical Characteristics of GC Patients

There were 233 cases in the LNM group, including 160 men and 73 women. Their median age was 63 years, their median BMI was 21.63 kg/m^2^, and their median tumor size was 4.5 cm. The positive CT findings were 136 cases and negative CT findings were 97 cases. There were 233 cases in the without LNM group, including 174 men and 59 women. Their median age was 62 years, their median BMI was 21.52 kg/m^2^, and their median tumor size was 3.0 cm. The positive CT findings were 54 cases and negative CT findings were 179 cases. There was no statistically significant difference in age (*P* = 0.44), sex (*P* = 0.15), BMI (*P* = 0.332), or tumor location (*P* = 0.146) between the two groups (Table 1).

**Table 1:** General characteristics of patients with GC and comparison of the relevant factors between the two groups.

**Factors**	**LNM (*n* = 233)**	**Without LNM (*n* = 233)**	***P*-value**
Age (years)	63 (54, 69)	62 (54, 68)	0.44
Sex (*n*)			0.15
Male	160	174	
Female	73	59	
BMI (kg/m^2^)	21.63 (19.15, 23.36)	21.51 (19.81, 23.51)	0.332
Tumor size (cm)	4.5 (3.5, 6.5)	3.0 (1.8, 4.0)	0.000
Tumor location (*n*)			0.146
Fundus of stomach	39	33	
Body of stomach	67	57	
Antrum of stomach	127	143	
Histological grade (*n*)			0.000
Low	122	88	
Low-medium	66	70	
Medium	45	69	
Medium-high	0	6	
CT findings (*n*)			0.000
Node positive	136	54	
Node negative	97	179	
Neutrophil (10^9^/L)	3.56 (2.77, 4.71)	3.55 (2.80, 4.50)	0.862
Lymphocyte (10^9^/L)	1.48 (1.13, 1.83)	1.53 (1.16, 1.84)	0.536
Hemoglobin (g/L)	122 (107, 134)	131 (116.5, 143.5)	0.000
Platelet (10^9^/L)	234 (183, 288.5)	214 (172, 266.5)	0.038
Albumin (g/L)	39.03 ± 3.91	40.43 ± 3.57	0.000
Fibrinogen (g/L)	3.10 (2.50, 3.83)	2.80 (2.41, 3.44)	0.014
CEA (ng/ml)	2.81 (1.70, 5.53)	2.55 (1.70, 3.92)	0.029
CA19-9 (U/ml)	14.57 (8.88, 29.49)	12.09 (8.24, 18.14)	0.000
CA125 (U/ml)	10.03 (6.80, 14.75)	8.80 (6.00, 12.98)	0.003
CA72-4 (IU/ml)	3.28 (1.44, 5.57)	2.06 (1.14, 4.63)	0.008

### Univariate and Multivariate Analysis

Univariate analysis found that the factors associated with LNM in GC were tumor size (*P* = 0.000), histological grade (*P* = 0.000), CT findings (*P* = 0.000), Hb (*P* = 0.001), platelets (*P* = 0.038), albumin (*P* = 0.000), fibrinogen (*P* = 0.014), CEA (*P* = 0.029), CA19-9 (*P* = 0.000), CA125 (*P* = 0.003), and CA72-4 (*P* = 0.008) (Table 1). Multivariate analysis showed that the independent risk factors for LNM in GC were tumor size (*OR*: 2.058; *P* = 0.000), CT findings (*OR*: 1.969; *P* = 0.000), grade (*OR*: 0.479; *P* = 0.000), Hb (*OR*: 1.211; *P* = 0.005), CEA (*OR*: 1.111; *P* = 0.017), and CA19-9 (*OR*: 1.040; *P* = 0.033) (Table 2).

**Table 2:** Risk factors of LNM in GC for multivariate conditional logistic regression analysis.

**Risk factors**	**B**	**SE**	**Wals**	***P*-value**	**OR (95%CI)**
Tumor size	1.164	0.226	26.616	0.000	2.058 (3.203, 4.985)
CT findings	1.120	0.226	24.648	0.000	1.969 (3.064, 4.768)
Grade	−0.478	0.131	13.267	0.000	0.479 (0.620, 0.802)
Hemoglobin	0.628	0.223	7.950	0.005	1.211 (1.874, 2.900)
CEA	0.581	0.243	5.727	0.017	1.111 (1.788, 2.879)
CA19-9	0.499	0.235	4.528	0.033	1.040 (1.648, 2.610)

### ROC Curve

**Figure 1:**
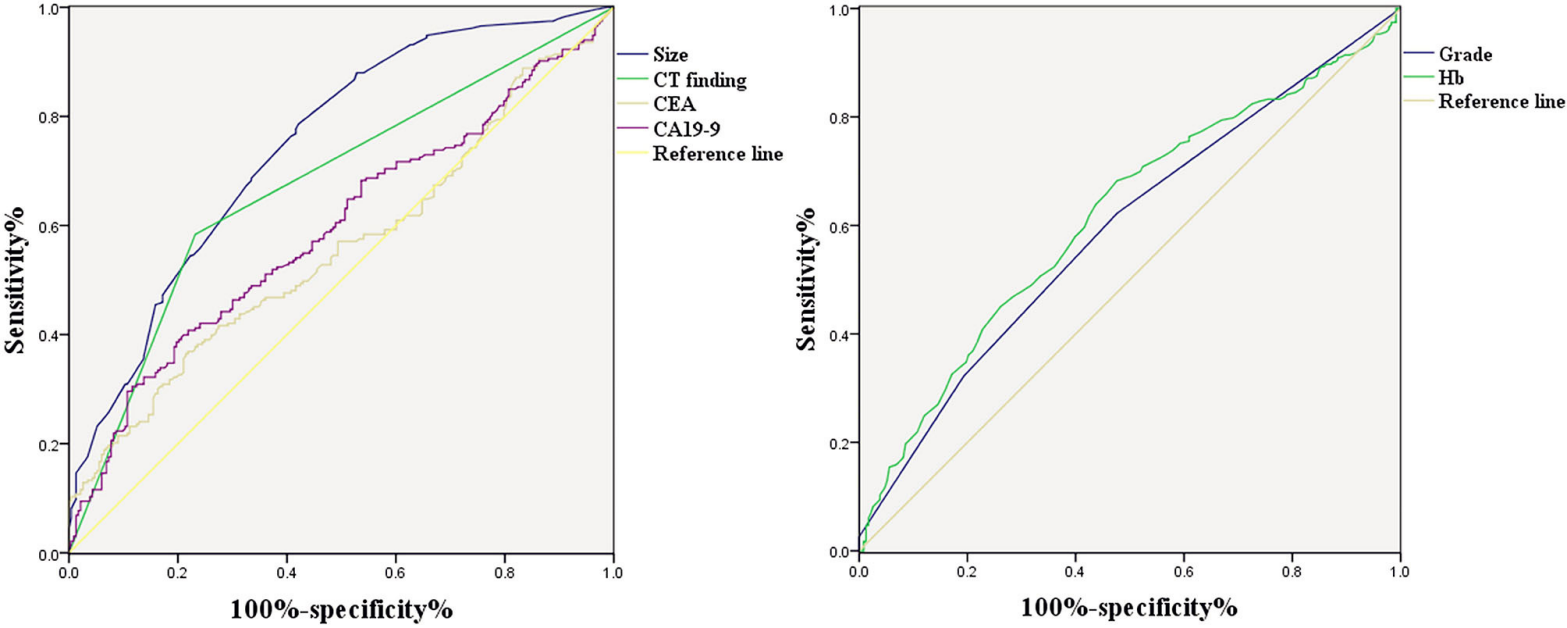
ROC curve of independent risk factors of LNM in GC, including CT finding, CEA, CA19-9, grade, and Hb.

Figure 1 shows the correlations between LNM of GC and tumor size, CT findings, grade, Hb, CEA, and CA19-9. According to the ROC curve evaluation, the optimal cut-off values for tumor size, Hb, CEA, and CA19-9 were 3.1 cm, 122.5 g/L, 4.285 ng/ml, and 19.19 U/ml, respectively. The area under the curve (AUC) for tumor size was 0.745, the 95% CI was 0.701–0.789, the sensitivity was 78.5%, and the specificity was 57.9%. The AUC for CT findings was 0.676, the 95% CI was 0.627–0.725, the sensitivity was 58.4%, and the specificity was 76.8%. The AUC for grade was 0.592, the 95% CI was 0.541–0.643, the sensitivity was 62.2%, and the specificity was 52.4%. The AUC for CEA was 0.558, the 95% CI was 0.506–0.611, the sensitivity was 36.9%, and the specificity was 78.1%. The AUC for CA19-9 was 0.593, the 95% CI was 0.541–0.645, the sensitivity was 40.8%, and the specificity was 78.1% (Table 3). These results show that the accuracy and sensitivity of individual indicators for determining LNM were relatively low.

**Table 3:** Independent risk factors for LNM in GC of ROC curve.

**Factors**	**AUC**	**95% CI**	**Sensitivity (%)**	**Specificity (%)**	**Cut-off value**	***P*-value**
Tumor size	0.745	(0.701, 0.789)	78.5	57.9	3.1	0.000
CT finding	0.676	(0.627, 0.725)	58.4	76.8		0.000
Grade	0.592	(0.541, 0.643)	62.2	52.4		0.001
Hemoglobin	0.616	(0.565, 0.667)	68.2	52.4	122.5	0.000
CEA	0.558	(0.506, 0.611)	36.9	78.1	4.285	0.029
CA19-9	0.593	(0.541, 0.645)	40.8	78.1	19.19	0.001

### Random Forest Algorithm to Extract Important Risk Factors for LNM in GC

**Figure 2:**
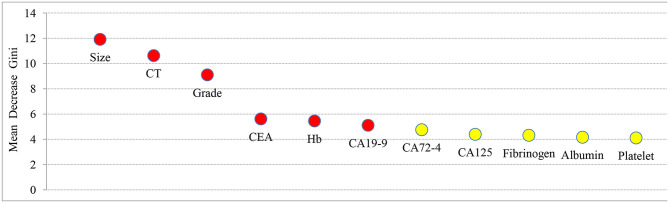
The importance ranking of factors related to LNM in GC, including tumor size, grade, CT findings, Hb, platelet, albumin, fibrinogen, CEA, CA19-9, CA125, and CA72-4. The larger the mean decrease of Gini, the more important the indicator was.

Factors related to LNM in GC, including tumor size, grade, CT findings, Hb, platelet, albumin, fibrinogen, CEA, CA19-9, CA125, and CA72-4, were analyzed by random forest algorithm, and the importance of variables was ranked (Figure 2). A larger mean decrease in Gini indicated the variable was more important. Tumor size ranked first and was followed by CT findings, grade, Hb, CEA, and CA19-9.

### Classification Tree Analysis to Obtain Decision Rules for the Preoperative Diagnosis of LNM in GC

**Figure 3:**
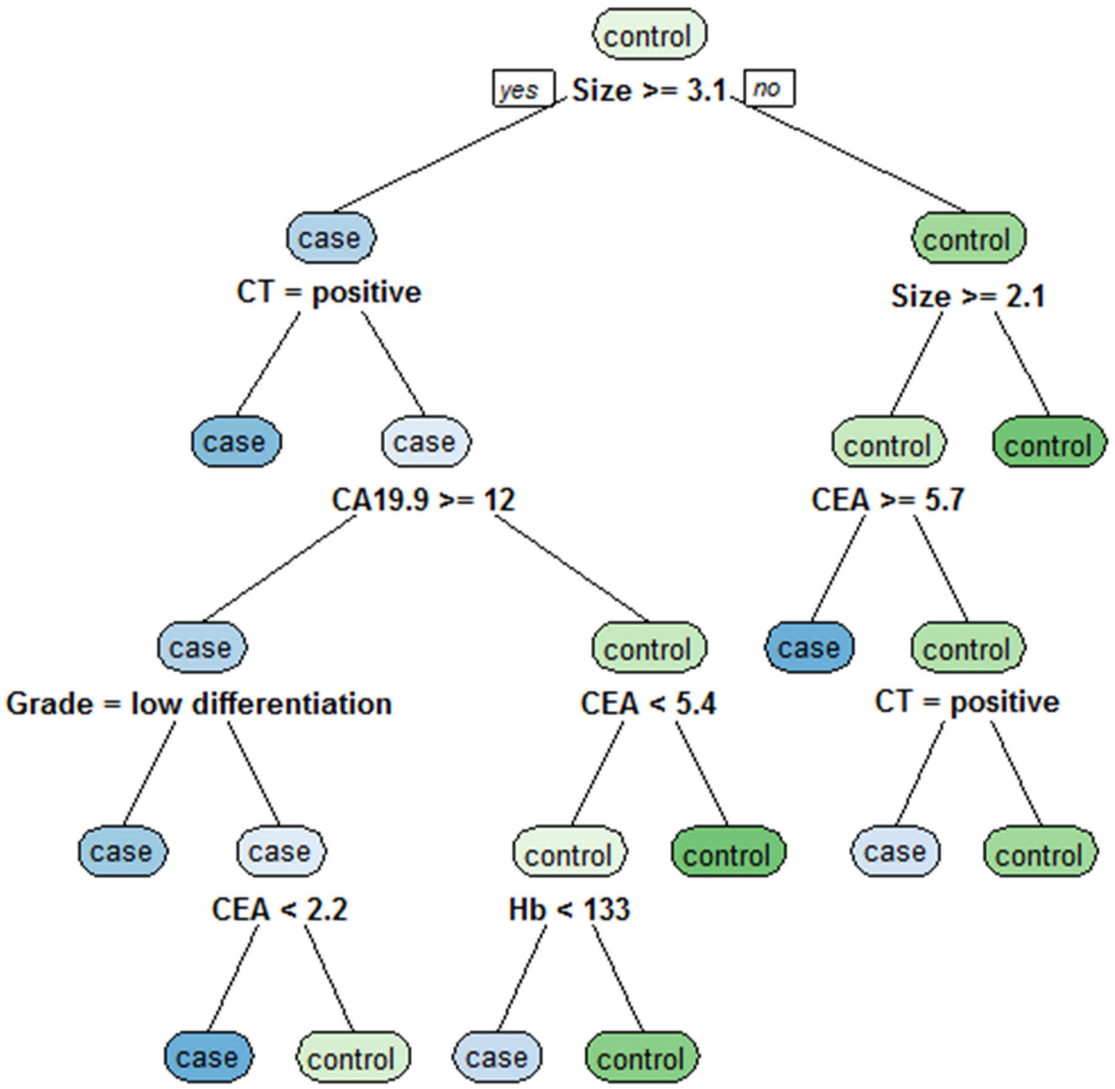
Classification tree for detecting undiagnosed LNM in GC, including tumor size, CT findings, grade, Hb, CEA, and CA19-9. Tumor size was the most important determinant because it was the first-level split of the two initial branches of the classification tree.

The independent risk factors for LNM in GC, including tumor size, CT findings, grade, Hb, CEA, and CA19-9, were analyzed by classification tree, and the decision rules affecting LNM in GC were obtained (Figure 3). Through the classification tree procedure, all variables were involved in the construction of decision rules for LNM in GC. Tumor size was the most important determinant because it was the first-level split of the two initial branches of the classification tree. CT findings were the most important determinant in the second-level split. The accuracy, sensitivity, specificity, positive predictive value and negative predictive value of the decision rules for diagnosing LNM in GC were 75.6, 85.7, 73.9, 73.5, and 79.3%, respectively, indicating that the decision rules were effective in the preoperative diagnosis of LNM in GC.

### Nomogram Model for Risk Assessment of Preoperative Diagnosis of LNM in GC

**Figure 4:**
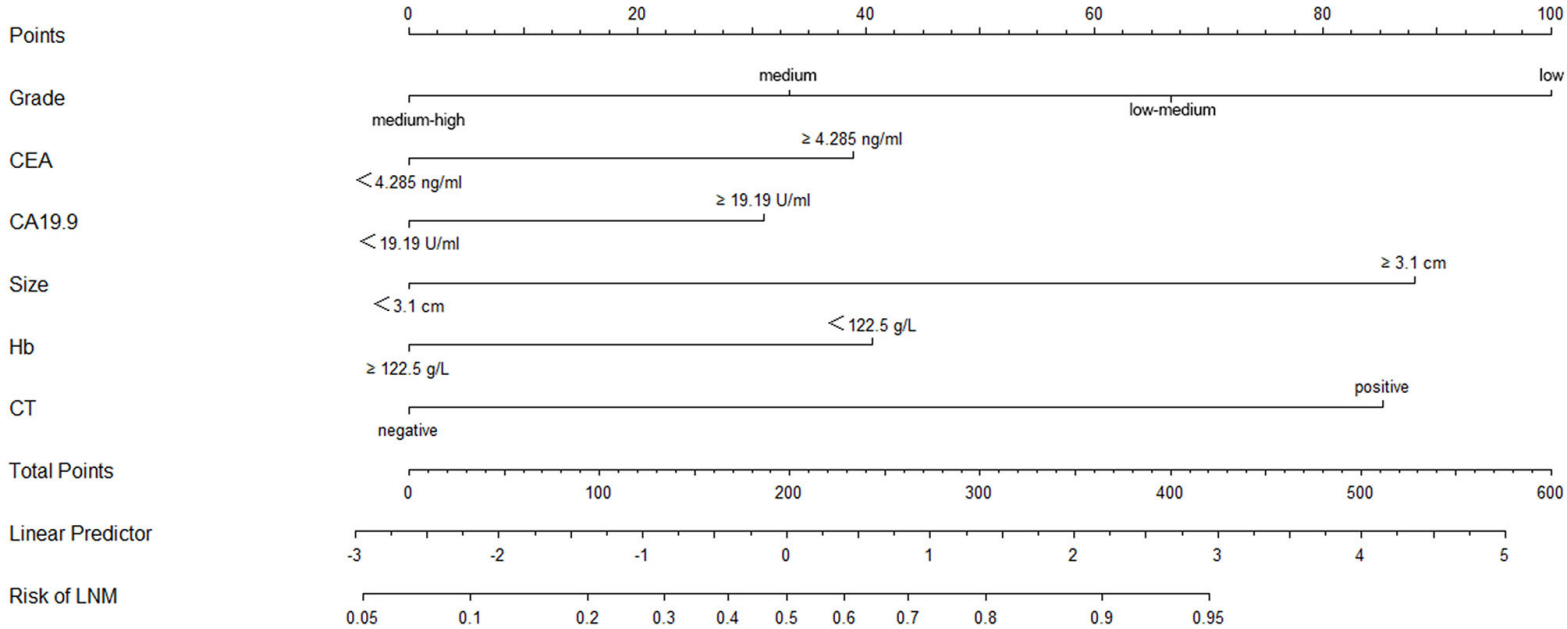
Nomogram of the logistic regression model constructed by grade, CEA CA19-9, tumor size, Hb, and CT findings. The points corresponding to the indicators were added to obtain the total points; the higher the total points, the higher the risk of LNM in GC.

**Figure 5:**
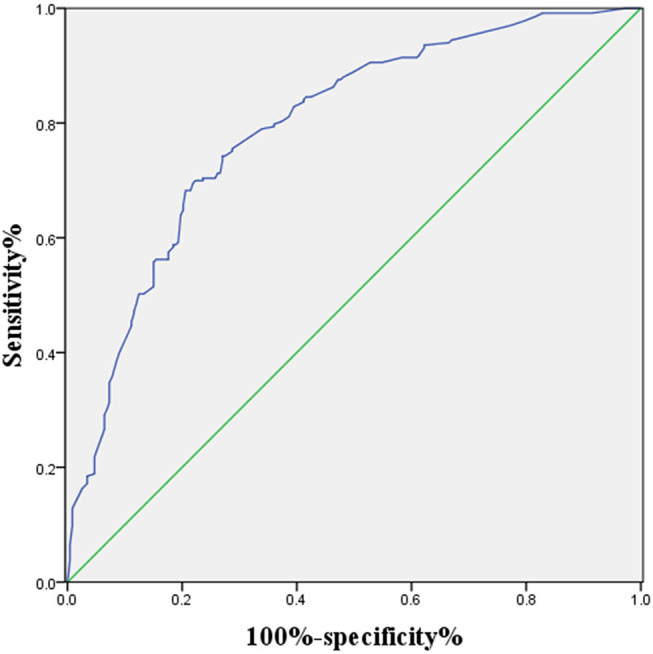
ROC curve of the combined factors (grade, CEA CA19-9, tumor size, Hb, and CT findings).

A logistic regression model that combined tumor size, CT findings, grade, Hb, CEA, and CA19-9 was constructed using the “rms” package in R language, and the C statistic of its evaluation was 0.790, indicating that the predictive model had high accuracy. Next, the plotting function was constructed, and the nomogram was plotted (Figure 4). The score for a tumor size ≥3.1 cm was 88 points, the score for a tumor size <3.1 cm was 0 points; the score for positive CT findings was 85 points; the score for negative CT findings was 0 points; the score for low differentiation was 100 points; the score for low-medium differentiation was 67 points; the score for medium differentiation was 33 points; the score for medium-high differentiation was 0 points; the score for hemoglobin ≥122.5 g/L was 0 points, the score for hemoglobin <122.5 g/L was 41 points; the score for CEA ≥4.285 ng/ml was 39 points, the score for CEA <4.285 ng/ml was 0 points; the score for CA19-9 ≥19.19 U/ml was 31 points, and the score for CA19-9 <19.19 U/ml was 0 points. The total score was 384, indicating that the probability of LNM in preoperative GC was 90–95%, indicating the risk of LNM in preoperative GC can be predicted based on the total points (Table 4). The AUC of the combined factors was 0.793, and the sensitivity and specificity were 80.3 and 79.4%, respectively (Figure 5), suggesting that this predictive model effectively predicted the risk of LNM in preoperative GC.

**Table 4:** Relationship between total points and risk of LNM in GC.

**Total points**	**Risk of LNM**
<32	<10%
32–93	11–20%
94–134	21–30%
135–168	31–40%
169–198	41–50%
199–229	51–60%
230–262	61–70%
263–303	71–80%
304–364	81–90%
365–421	91–95%
>421	>95%

## Discussion

LNM is an important indicator when evaluating the prognosis of GC (8, 24, 25). Radical surgery and adjuvant therapy are standard treatments for GC (4, 26). Post-operative prognosis is mainly assessed based on the TNM staging system of the American Joint Committee on Cancer (AJCC) (8, 9). However, this system only reflects the characteristics of the cancer itself, and it is difficult to obtain accurate data before surgery. In addition, accurate judgment of preoperative lymph node status is crucial for selecting treatment options for GC. Therefore, an accurate preoperative diagnosis of LNM is of great significance for the formulation of treatment plans and the evaluation of prognosis of patients with GC. After a series of analyses, we only used the indicators available before surgery, including tumor size, CT findings, grade, Hb, CEA, and CA19-9, to establish the decision rules and risk assessment model for LNM in GC.

The NCCN guidelines recommend the use of CT scan, EUS, PET/CT, and MRI to diagnose LNM in patients with GC before surgery (9, 27). With the continuous development of technology, MDCT has become the most commonly used technique for preoperative staging of GC. It provides specific value in the evaluation of infiltration depth, ascites, and distant metastasis but has limitations in the diagnosis of LNM (28, 29). Although improved CT analysis techniques, including those that take into account size and/or imaging patterns (enhancement, necrosis, shape, and fat content), were used to assess LNM, the overall accuracy was also approximately 60–80% (30, 31). One reason for this result is that small lymph nodes may contain metastatic tumors, while large lymph nodes may be caused by inflammation (32). The previous MDCT diagnostic criteria for LNM in GC were as follows (31, 33–35): (1) short axis >8 mm; (2) short axis (perigastric) >6 mm and short axis (extraperitoneal) >8 mm; (3) significantly enhanced long axis >8 mm; and (4) central necrosis or aggregation (three nodes or more), regardless of size. Moreover, some studies have suggested that any identifiable lymph nodes were positive (15, 36). The criteria used in this study to judge LNM were consistent with the latter. The results of LNM differed according to different criteria. Ohashi et al. found that the accuracy, sensitivity, and specificity of CE-MDCT in the diagnosis of LNM were 67.9, 79.1, and 50.0%, respectively (37). Hasegawa et al. (31) showed that the specificity of CT in the diagnosis of LNM was 96.8%, while the sensitivity was only 46.2%. A clinical trial conducted in recent years indicated that the sensitivity and positive predictive value of CT scans in the diagnosis of LNM were 62.5 and 77.7%, respectively (38). In this study, the accuracy, sensitivity, and specificity of CE-MDCT in the diagnosis of LNM were 67.6, 58.4, and 76.8%, respectively. Meanwhile, Lee et al. (39) showed that MDCT had some limitations in the determination of GC restaging after short-term NAC, and proposed that CT volumetry for primary gastric tumor can accurately predict the pathological reaction after NAC in AGC. Lundsgaard et al. (40) indicated that CT perfusion assessment of gastroesophageal junction carcinoma and GC showed moderate sensitivity and specificity in preoperative chemotherapy response, so it was not recommended for clinical decision purposes. Park et al. (41) demonstrated that after NAC for locally AGC, the accuracy of CT diagnosis of T and N staging were 57 and 37%, respectively. A randomized phase II study found that CT restaging after NAC was considered inaccurate and unreliable for GC (42). Recent studies (43) showed that CT radiomics—the high-throughput extraction of quantitative imaging features characterizing the spatial relationships and consistency of signal intensities—may make it possible to predict the adverse reactions after NAC for locally AGC.

In addition, the accuracy of MDCT in diagnosing LNM can also be influenced by the clinical experience of the radiologists. Therefore, there is a clinical need for objective indicators that predict LNM. Tumor size can, when based on the maximum diameter of the tumor, be measured using imaging techniques or endoscopy. Choi et al. (44) found that the tumor size measured by gastroscopy was very consistent with the tumor size determined by pathology, and in 90% of the patients, the absolute measurement difference was <0.6 cm. Zhao et al. (45) showed that there was no statistically significant difference between MSCT measurement of the maximum diameter and post-operative measurement results in GC, indicating that CT measurement of tumor maximum diameter can be used as a reliable method. Vilgrain et al. (46) demonstrated that the tumor size measured by sonography was correlated well with findings at surgery, and tumor length as seen on CT correlated with the macroscopic appearance of the lesion. In order to obtain the most accurate tumor size, it was measured from post-operative specimens in this study. Histological grade can be obtained by biopsy under a gastroscope before surgery. Tumor markers and hemoglobin are routine preoperative test items. In this study, multivariate analysis showed that tumor size, CT findings, grade, Hb, CEA, and CA19-9 were independent risk factors for LNM in GC, and tumor size was found to be the most important factor for the evaluation of LNM of GC through a random forest algorithm and classification tree. Previous studies have shown that tumor size is significantly associated with LNM in GC (47–49). A larger tumor will have a higher risk of LNM (50, 51). Habermann et al. (52) and Isomoto et al. (53) proposed that tumor size, depth of invasion, and differentiation degree are important clinicopathological factors that should be considered to accurately determine the risk of LNM in GC. In EGC, tumor size, depth of invasion, lymphatic infiltration, gross type, and histologic type were related to LNM (54–56). Nakagawa et al. (57) retrospectively analyzed the clinical data of 1,042 EGC patients undergoing gastrectomy and lymph node dissection and found that age, tumor size, the depth of invasion, the presence of ulcers, and positive CT findings were high-risk predictors of LNM. Lin et al. (58) retrospectively analyzed the clinical data of 1,460 EGC patients in multiple centers and found that women, tumors larger than 20 mm, submucosal invasion, and undifferentiated tumor histological types were independent risk factors for LNM. Xu et al. (59) believed that combining MDCT, preoperative histological type and tumor size was an effective method to predict LNM in GC.

Tumor markers are also related to LNM in tumors. Preoperative elevated serum CEA and CA-153 levels are risk factors for axillary LNM in patients with breast cancer (60). CA724, CA199, and CEA are significantly correlated with LNM in GC and can be used as reliable biomarkers for predicting LNM in GC (61–63). Duraker and Celik (62) showed that CEA has value in the diagnosis of LNM in GC. Previous studies demonstrated that elevated levels of CA125 are associated with GC peritoneal dissemination and a poor prognosis (64, 65). In this study, univariate analysis found that CEA, CA19-9, CA125, and CA72-4 were associated with LNM in GC, and multivariate analysis showed that CEA and CA19-9 were independent risk factors for LNM in GC. Hb is an indicator of anemia. Hypoxia caused by anemia may accelerate tumor angiogenesis to promote tumor invasion and progression (66, 67). In addition, hypoxia also increases the expression of hypoxia-inducing factor 1 (HIF-1), which regulates gene products that can promote tumor invasion and metastasis (68). Lysyl oxidase (LOX) is also a hypoxic gene that is involved in the migration and invasion of cancer cells (69). Hypoxia may increase the potential for proliferation and metastasis by inducing proteomic and genomic changes (66). Previous studies found that anemia was related to a poor prognosis in patients with GC (70, 71). Shen et al. (72) showed that preoperative anemia was significantly correlated with large tumors, deep infiltration, and high tumor stage. In this study, we found that low Hb levels were a high-risk predictor of LNM in GC. Previous studies have also identified new molecular biomarkers for predicting LNM and successfully identified several markers for predicting LNM through proteomics and histological analysis, such as miR-1207-5p, LYVE-1 antibody, 14-3-3β, profilin-1 protein, inhibitors of cytokine signaling 3, p53 gene, Ki67, EGFR, and miR-1207-5p (17, 18, 73–76). However, due to their high cost, complicated technology, and difficult preoperative detection, their clinical applications are limited.

A phase II trial demonstrated that NAC can improve the R0 resection rates in stage II or III GC patients with LNM, and showed that the 3-year relapse-free survival was 44.9%, and the 3-year overall survival rate was 48.0% (77). Tsuburaya et al. (11) indicated that for GC with widespread LNM, NAC followed by surgery can improve patient survival time. Sym et al. (78) found that for GC with para-aortic LNM, NAC can significantly improve the surgical resection rate. Meanwhile, a randomized controlled trial showed that for operable GC patients, including 93 patients with LNM, NAC can reduce tumor size and stage, and significantly improve progression-free survival and overall survival (13). Inoue et al. (79) found that in the seven GC patients with T4N1M0 tumor who underwent R0 resection after NAC, no patient had local relapse during the long-term follow-up. Therefore, they believed that NAC can improve the prognosis of GC patients with T4N1M0 tumor.

Nomograms have been widely used to quantify the risk factors for LNM in various cancers (19, 20). Recently, nomograms were also developed to predict LNM in GC (21, 80). However, some of the factors they used were available only after surgery, reducing their clinical value. In this study, we obtained six preoperative factors through analyses of routine examinations and developed a decision rules and risk assessment model for LNM in GC. The accuracy, sensitivity, and specificity of the decision rules in diagnosing preoperative LNM in GC were 75.6, 85.7, and 73.9%, respectively. The accuracy, sensitivity, and specificity of the risk assessment model in predicting preoperative LNM in GC were 79.3, 80.3, and 79.4%, respectively. These results indicate that these factors can effectively predict the incidence of LNM in preoperative GC. This study has some limitations. First, this was a single-center retrospective study. Second, the sample size was not large. Third, the established decision rules and nomogram models have clinical value in the diagnosis of LNM in GC but are limited in the ability to accurately predict the number of LNMs. Finally, tumor size was measured by post-operative specimens. Therefore, multicenter large-scale prospective randomized controlled trials are necessary.

## Conclusion

In this study, we used only the indicators available before surgery to establish decision rules and a risk assessment model for LNM in GC and found that tumor size, CT findings, grade, Hb, CEA, and CA19-9 were independent risk factors for LNM in GC. Further analysis showed that tumor size was the most important factor in evaluating LNM in GC. The decision rules and nomogram model constructed to use tumor size, CT findings, grade, Hb, CEA, and CA19-9 cam effectively predict the incidence of LNM in preoperative GC and thereby provide a reference value for the formulation of treatment plans and the evaluation of prognosis in patients with GC.

## Data Availability Statement

The raw data supporting the conclusions of this article will be made available by the authors, without undue reservation.

## Ethics Statement

The studies involving human participants were reviewed and approved by Second Affiliated Hospital of Nanchang University. The patients/participants provided their written informed consent to participate in this study.

## Author Contributions

CHua designed the study, analyzed the data, and wrote the manuscript with contributions from all authors. CHua, CHu, JZ, and WZ collected the clinical data. JH and ZZ provided critical comments for this paper. All authors read and approved the final version of the paper.

## Conflict of Interest

The authors declare that the research was conducted in the absence of any commercial or financial relationships that could be construed as a potential conflict of interest.
